# Incidence and risk factors for hyperglycemia in pregnancy among nulliparous women: A Brazilian multicenter cohort study

**DOI:** 10.1371/journal.pone.0232664

**Published:** 2020-05-13

**Authors:** Bianca F. Nicolosi, Renato T. Souza, Jussara Mayrink, Francisco E. Feitosa, Edilberto A. Rocha Filho, Débora F. Leite, Janete Vettorazzi, Maria H. Sousa, Maria L. Costa, Philip N. Baker, Louise C. Kenny, Jose G. Cecatti, Iracema M. Calderon, for the Preterm SAMBA Study Group

**Affiliations:** 1 Department of Obstetrics and Gynecology, Botucatu Medical School, Unesp, Botucatu, SP, Brazil; 2 Department of Obstetrics and Gynecology, University of Campinas (UNICAMP) School of Medical Sciences, Campinas, SP, Brazil; 3 MEAC–Maternity School of the Federal University of Ceará, in Fortaleza, CE, Brazil; 4 Department of Maternal and Child Health, Maternity of Clinic Hospital, Federal University of Pernambuco, Recife, PE, Brazil; 5 Department of Obstetrics and Gynecology, Maternity of the Clinic Hospital, Federal University of RS, Porto Alegre, RS, Brazil; 6 Statistics Unit, Jundiai School of Medicine, Jundiaí, SP, Brazil; 7 College of Life Sciences, University of Leicester, Leicester, United Kingdom; 8 Faculty of Health and Life Sciences, Department of Women’s and Children’s Health, Institute of Translational Medicine, University of Liverpool, Liverpool, United Kingdom; Universidade Federal do Rio de Janeiro, BRAZIL

## Abstract

**Objective:**

To assess the incidence and risk factors for hyperglycemia in pregnancy in a cohort of Brazilian nulliparous pregnant women.

**Materials and methods:**

This is a secondary analysis of a multicenter cohort study that enrolled 1,008 nulliparous pregnant women at 19–21 weeks. Exclusion criteria included chronic exposure to corticosteroids and previous diabetes. Bivariate and multivariate analyses by Poisson regression were used to identify associated factors.

**Results:**

The incidence of hyperglycemia in pregnancy was 14.9% (150/1,008), and 94.7% of these cases were gestational diabetes mellitus (142/150). Significant associated factors included a family history of diabetes mellitus, maternal overweight or obesity at enrollment, and previous maternal conditions (polycystic ovarian syndrome, thyroid dysfunctions and hypertensive disorders). A BMI ≥ 26.3Kg/m^2^ (RR_adj_ 1.87 [1.66–2.10]) and a family history of diabetes mellitus (RR_adj_ 1.71 [1.37–2.15]) at enrollment were independent risk factors for HIP.

**Conclusions:**

A family history of diabetes mellitus and overweight or obesity (until 19–21 weeks of gestation) may be used as selective markers for HIP in Brazilian nulliparous women. Given the scarcity of results in nulliparous women, our findings may contribute to determine the optimal diagnostic approach in populations of similar socioeconomic characteristics.

## Introduction

The International Association of Diabetes and Pregnancy Study Group (IADPSG) and the International Federation of Gynecology and Obstetrics (FIGO) divided hyperglycemia in pregnancy (HIP into two distinct conditions: Diabetes in pregnancy (DIP) and Gestational Diabetes Mellitus (GDM). DIP is defined as diabetes diagnosed before pregnancy o hyperglycemia with first recognition during pregnancy according to WHO diagnostic criteria for non-pregnant women that may occur at any time during pregnancy including the first trimester. GDM is defined as pregnancy related hyperglycemia (other than DIP) OR hyperglycemia with first recognition during pregnancy that may also occur at any time during pregnancy, but most likely occurs after 24 weeks of gestation [1–4].

According to the FIGO, HIP is one of the most common complications in pregnancy due to the epidemic of obesity and diabetes, also referred to as the DIABESITY epidemic. HIP is estimated to affect one in six pregnant women and 84% of them are GDM. Brazil is one of the eight low- and middle-income countries contributing to 55% of global live births and 55% of the global burden of diabetes [1–4]. According to the Brazilian Gestational Diabetes Study (EBDG), a multicenter cohort that included 5,564 Brazilian pregnant women, the estimated prevalence of GDM was 18% according to the IADPSG criteria [5].

A recent Brazilian consensus recommended universal screening with fasting plasma glucose (FPG) and a 75-g oral glucose tolerance test (OGTT) in settings where technical and financial resources are available, identifying 100% of GDM cases. In suboptimal settings, a normal FPG (< 92 mg/dL) at the first antenatal visit, and screening repeated at 24–28 weeks of gestation can identify 86% of GDM cases [6].

The previous consensus document from WHO initially led to a policy of universal screening [3], however, this resulted in the diagnosis of GDM in a growing number of women, without sufficient evidence of improvement in maternal/neonatal outcomes or cost-effectiveness. Therefore, controversy persists over whether to screen for GDM. Several clinical and biomolecular risk factors have already been tested, either alone or in algorithms. The predictive performance of these markers has actually been poor, due to low prevalence rates and population-dependent variations in risk factors. Furthermore, there is a diversity of suggested diagnostic criteria for GDM across studies [7–9].

The identification of risk factors in a particular population of pregnant women, along with a well-defined diagnostic protocol, may improve the performance of risk factors in predicting HIP (DIP or GDM). Although several studies have previously identified classical risk factors for GDM, relatively few studies have been conducted in nulliparous women [10–12], and none were focused on Brazilian healthy nulliparous women. Our objective was to assess the incidence and risk factors for HIP (DIP or GDM) in a cohort of Brazilian nulliparous pregnant women.

## Materials and methods

This is secondary analysis of the Preterm SAMBA, a prospective multicenter cohort study conducted from July 2015 to July 2018 in five Brazilian obstetric referral centers: the University of Campinas (UNICAMP)/SP, Botucatu Medical School, Unesp/SP, Federal University of Ceará (UFC)/CE, Federal University of Pernambuco (UFP)/PE, and Federal University of Rio Grande do Sul (UFRGS)/RS. The study protocol was approved by the Institutional Review Board (IRB) of the School of Medical Sciences of The University of Campinas (Letter of approval 1.048.565 issued on 28th April 2015), and all other Brazilian participating centers: IRB from the Maternidade Escola Assis Chateaubriand of the Federal University of Ceara in Fortaleza, IRB from the Center for Health Sciences of the Federal University of Pernambuco in Recife, IRB from the Clinics Hospital of the Federal University of Rio Grande do Sul in Porto Alegre, and the IRB from the Clinics Hospital of Botucatu Medical School at the University of the State of Sao Paulo (Unesp) and amended by the Brazilian National Committee for Ethics in Research (CONEP). The study complies with national and international regulations for experiments in human beings, including resolution CNS 466/12 of the Brazilian National Heath Council and the 1989 Declaration of Helsinki. Each woman signed an informed consent form before entering the study. This manuscript follows the Strengthening the Reporting of Observational studies in Epidemiology (STROBE) Statement [13].

Methodological details and operational procedures of the Preterm SAMBA study had already been published elsewhere [14–16]. Briefly, the preterm SAMBA project was divided into two phases [14–16]: 1) discovery of a predictive model based on data and samples from an international multicenter cohort entitled SCOPE study (which included only nulliparous women) [16]; 2) validation of the predictive model using a multicenter Brazilian cohort. Matching the eligibility criteria of participants from the SCOPE and the Preterm SAMBA studies was crucial for developing and validating the predictive model. The validation of predictive model, however, is not the scope of the current analysis. In addition to preterm birth, other major pregnancy complications have been considered as secondary outcomes for the Brazilian cohort, including hyperglycemia in pregnancy, preeclampsia, and fetal growth restriction.

### Subjects

The study enrolled nulliparous singleton pregnant women from 19+0 to 21weeks of gestation. Exclusion criteria included chronic exposure to corticosteroids and previous type 1 or type 2 Diabetes Mellitus (T1DM or T2DM) and other maternal chronic diseases and use of medications/supplements [14].

### Sample size

The sample size was calculated according to spontaneous preterm birth outcome, which was the main outcome for the cohort. Assuming a type I error of 5%, accuracy of at least 0.68 for the test measured by the area under the ROC curve, and adequate power (80% of power, β = 0.2) to test the hypothesis, the sample size should approach 80 cases of preterm births. Considering that the expected minimum prevalence of preterm birth is 7% in Brazil, the sample size calculated was 1,150 women. Estimating a prevalence of around 10–15% of GDM in nulliparous pregnant women [17], the Preterm SAMBA study population would be able to identify about 115 to 170 cases.

### Data collection procedures

Eligible women were identified in the primary health care units and in the obstetric antenatal clinics in the referral maternities. Women were included between 19 and 21 weeks of gestation (first study visit), and a comprehensive assessment was conducted to gather information on sociodemographic characteristics, reproductive history, personal and family medical history. After the interview, anthropometric and clinical measurements, and a nutritional assessment based on a 24-hour diet recall were performed according to standardized protocols. The same clinical andanthropometric evaluation was also performed during two subsequent study visits (at 27–29 and 37–39 weeks of gestation). A review of the medical record and prenatal chart was conducted in the postpartum period to collect maternal and newborn information related to the late pregnancy, intrapartum and postpartum periods, in addition to newborn data [14]. The collected data had been entered in an online database system.

Data regarding the results of fasting plasma glucose from early- to mid-pregnancy was recorded at the 19–21 weeks study visit. Data on OGTT or fasting plasma glucose dones in the second half of pregnancy was recorded during the second and third study visits (27–29 and 37–37 weeks) and during the postpartum medical record review. Although the diagnostic protocol for HIP was previously recommended [1–4], each center employed its own diagnostic and treatment protocol, according to physical, structural and economic conditions, as recommended by the Brazilian guidelines [6]. Data were entered into a central database accessible through the Internet, provided with a complete audit trail (MedSciNet®).

### Outcome

In the current study, the outcome was hyperglycemia in pregnancy (HIP). It was divided into two distinct forms: diabetes in pregnancy (DIP) and gestational diabetes mellitus (GDM) [1–4]. DIP was defined as diabetes diagnosed before pregnancy or hyperglycemia with first recognition during pregnancy according to WHO diagnostic criteria for non-pregnant women, and diagnosed by fasting plasma glucose ≥ 7.0 mmol/L (126 mg/dL) or 2-hour plasma glucose ≥ 11.1 mmol/L (≥ 200 mg/dL) following a 75g OGTT or a random plasma glucose ≥ 11.1 mmol/L (≥ 200 mg/dL) with diabetes symptoms [1–4]. GDM was defined as hyperglycemia (other than DIP) during pregnancy or hyperglycemia with first recognition during pregnancy. GDM diagnostic criteria were fasting plasma glucose ≥ 5.1 and ≤ 6.9 mmol/L (≥ 92 and ≤ 125 mg/dL) or 1-hour plasma glucose ≥ 10.0 mmol/L (≥ 180 mg/dL) following a 75g OGTT or a 2-hour plasma glucose ≥ 8.5 and ≤ 11.0 mmol/L (≥ 153 and ≤ 199 mg/dL) following a 75g OGTT [1–4].

### Risk factors associated with HIP

The following sociodemographic and maternal clinical characteristics were addressed as potential risk factors for HIP: maternal age ≥ 25 years, non-white ethnicity, marital status (without a partner), schooling < 12 years, lower annual family income, source of prenatal care (public health services), reproductive and family history of diabetes–first-degree relatives with DM and pregnant woman whose mother had GDM during her pregnancy, smoking and alcohol habits, maternal weight gain (WG) at 20–27 weeks of gestation, body mass index (BMI) at study enrollment, any previous disorders (polycystic ovarian syndrome, thyroid dysfunction, previous hypertensive disorder), and blood pressure at 20 weeks of gestation were evaluated. Likewise, we evaluated some maternal and neonatal outcomes commonly described for women with HIP [7–9].

The proportion of women in each quartile (below Q1, Q1-Q2, Q2-Q3 and above Q3) and percentile category (< p10, p10-p90, and > p90) of weight gain per week between the first and second visit were also addressed for both HIP and control groups. Due to the difficulty in obtaining information on pre-gestational weight, maternal weight at the first visit (19–21 weeks of gestation) was defined as the reference for estimation of WG from 20 to 27 weeks of gestation and BMI at enrollment, classified according to the new references for Brazilian pregnant women [18].

### Statistical analysis

Initially, we determined the incidence of HIP, the absolute and relative incidence of its components (DIP and GDM), and the frequency of abnormal 75g-OGTT results to offer treatment. To compare HIP and control (Non-HIP), we assessed potential risk factors, along with associated maternal and neonatal outcomes. A bivariate analysis was carried out to estimate Risk Ratios (RR) and their respective 95% Confidence Intervals (95%CI). Finally, a multivariate analysis was performed, using Poisson multiple regression with backward selection, to identify which factors were independently associated with HIP and estimate the adjusted RR (RR_adj_). Data analysis was adjusted for the Primary Sampling Unit (PSU) of the five centers/hospitals (*p* < .05). We used SPSS v20.0 and Stata v7.0 software.

## Results

**Fig 1:**
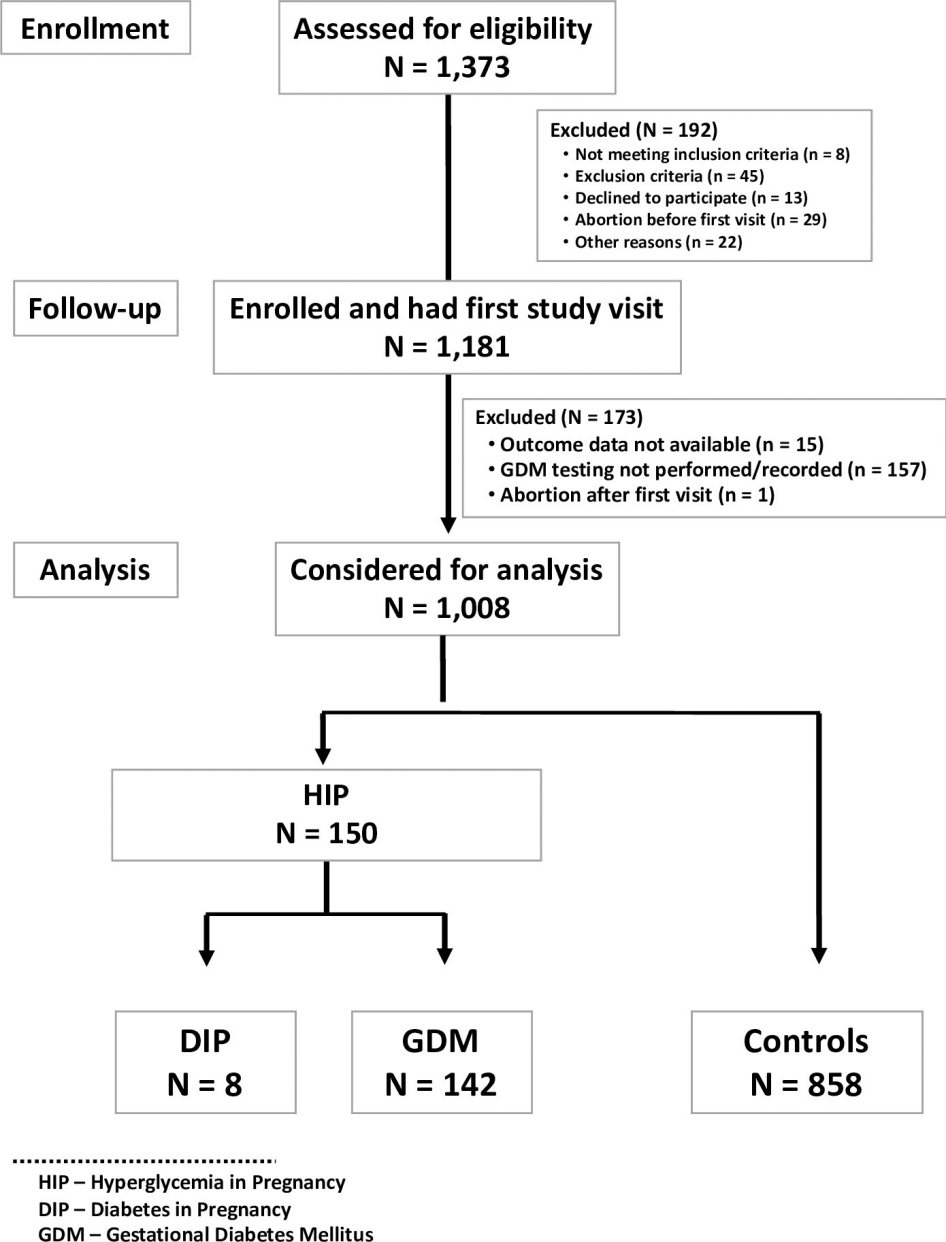
Flow chart of participating women in the study.

Fig 1 shows the study flow chart defined according to outcome–Hyperglycemia diagnosed during pregnancy (HIP), comprising Diabetes in Pregnancy (DIP) and Gestational Diabetes Mellitus (GDM). Table 1 shows the incidence of HIP (14.9%), with their components–DIP (0.8%) and GDM (14.1%), in Brazilian low-risk, nulliparous pregnant women, included in the Preterm SAMBA cohort. Of the 150 pregnant women diagnosed with HIP, 58 (38.7%) received no treatment and 92 (61.3%) were treated with diet and exercise alone (21.7%) or received adjuvant drugs (insulin or metformin) (78.3%).

**Table 1:** Diagnosis of hyperglycemia in pregnancy (HIP) in nulliparous Brazilian cohort study.

Incidence	n/N	Percent (%)
**HIP**	**150/1008**	**14.9**
GDM/HIP	142/150	94.7
DIP/HIP	8/150	5.3
**GDM**	**142/1008**	**14.1**
**DIP**	**8/1008**	**0.8**
**Treatment**		
**No**	**58/150**	**38.7**
Yes	92/150	61.3
** Diet and exercise alone**	**72/92**	**78.3**
** Drugs (insulin or metformin)**	**20/92**	**21.7**

HIP = Hyperglycemia in Pregnancy; GDM = Gestational Diabetes Mellitus; DIP = Diabetes in Pregnancy

Family history of DM [RR = 1.86; 1.50–2.30], overweight [RR = 1.49; 1.27–1.76], obesity [RR = 2.16; 1.57–2.96], and previous disorders (POS, thyroid dysfunction or hypertension) [RR = 1.81; 1.05–3.13] were significantly associated with the occurrence of HIP (Tables 2 and 3). In this cohort, maternal or perinatal outcomes were not significantly different in the HIP group as compared to the Control group (Table 4).

**Table 2:** Estimated risk of sociodemographic maternal characteristics for HIP.

Characteristics	HIP	Control	RR (95%CI)
Maternal age (years)			
< 25	56 (37.3)	481 (56.1)	Ref.
≥ 25	94 (62.7)	377 (43.9)	1.91 [0.85–4.33]
Ethnicity			
White	61 (40.7)	354 (41.3)	Ref.
Non-white	89 (59.3)	504 (58.7)	1.02 [0.70–1.50]
Marital status^(1)^			
With a partner	117 (78.5)	621 (72.5)	Ref.
Without a partner	32 (21.5)	235 (27.5)	0.76 [0.48–1.20]
Schooling (years)			
< 12	97 (64.7)	568 (66.2)	Ref.
≥ 12	53 (35.3)	290 (33.8)	1.06 [0.74–1.51]
Annual Family Income (US$)			
Up to 3000	6 (4.0)	37 (4.3)	0.91 [0.49–1.70]
3000 to 6000	75 (50.0)	438 (51.0)	0.96 [0.67–1.37]
Above 6000	69 (46.0)	383 (44.6)	Ref.
Source of prenatal care			
Entirely public	132 (88.0)	728 (84.8)	1.26 [0.90–1.76]
Private/insurance/mixed	18 (12.0)	130 (15.2)	Ref.
Total	**150**	**858**	

HIP = Hyperglycemia in Pregnancy

^(1)^ Missing = 3 cases

**Table 3:** Estimated risk of maternal lifestyle habits and characteristics for HIP.

Characteristics	HIP	Controls	RR (95%CI)
Mother history of GDM ^(1)^			
Yes	8 (6.0)	27 (3.4)	1.63 [0.80–3.33]
No	125 (94.0)	765 (96.6)	Ref.
Family history of DM			
Yes	47 (31.3)	152 (17.7)	**1.86 [1.50–2.30]**
No	103 (68.7)	706 (82.3)	Ref.
Smoking			
No smoking	138 (92.0)	793 (92.4)	Ref.
Ceased during pregnancy/current smoker	12 (8.0)	65 (7.6)	1.05 [0.41–2.69]
Alcohol drinking ^(2)^			
No alcohol	111 (86.7)	617 (81.8)	Ref.
Ceased during pregnancy/current drinker	17 (3.3)	137 (18.2)	0.72 [0.48–1.09]
Previous abortion			
Yes	17 (11.3)	97 (11.3)	1.00 [0.39–2.57]
No	133 (88.7)	761 (88.7)	Ref.
WG/week (kg)– 20 to 27 weeks ^(3)^			
<Q 1 (g)	42 (33.1)	174 (24.2)	1.37 [0.77–2.43]
Q1-Q2 (g)	29 (22.8)	175 (24.3)	Ref.
Q2-Q3 (g)	31 (24.4)	185 (25.7)	1.01 [0.65–1.56]
≥ Q3 (g)	25 (19.7)	186 (25.8)	0.83 [0.48–1.45]
Body Mass Index at enrollment^#^			
Underweight (< 21.5 kg/m^2^)	12 (8.0)	150 (17.5)	0.64 [0.17–2.40]
Normal weight (21.5–26.2)	47 (31.3)	356 (41.5)	Ref.
Overweight (26.3–30.9)	46 (30.7)	218 (25.4)	**1.49 [1.27–1.76]**
Obesity (> 30.9 kg/m^2^)	45 (30.0)	134 (15.6)	**2.16 [1.57–2.96]**
Any previous maternal disorder*			
No	103 (68.7)	702 (81.8)	Ref.
Yes	47 (31.3)	156 (18.2)	**1.81 [1.05–3.13]**
Blood pressure (BP) at 20^th^ week			
BP ≥ 140 x 90 mmHg	9 (6.0)	28 (3.3)	168 [0.88–3.19]
BP < 140 x 90 mmHg	141 (94.0)	830 (96.7)	Ref.
Blood pressure (BP) at 20^th^ week			
BP ≥ 130 x 85 mmHg	21 (14.0)	87 (10.1)	1.36 [0.87–2.11]
BP < 130 x 85 mmHg	129 (86.0)	771 (89.9)	Ref.
**Total**	**150**	**858**	

HIP = Hyperglycemia in Pregnancy; GDM = Gestational Diabetes Mellitus; DM = Diabetes Mellitus; WG = weight gain; SBP = Systolic Blood Pressure; DBP = Diastolic Blood Pressure

*Polycystic Ovarian Syndrome (POS) OR Thyroid dysfunctions OR Previous hypertensive disorder without medication

^(1)^ Missing = 83

^(2)^ Missing = 126

^(3)^ Missing = 161 cases

**Table 4:** Maternal and neonatal outcomes associated with HIP.

Outcomes	HIP	Controls	RR (95%CI)
Mother			
Mode of delivery			
Vaginal	70 (46.7)	465 (54.2)	Ref.
C-section with labor	40 (26.7)	197 (23.0)	1.30 [0.69–2.42]
C-section without labor	40 (26.7)	196 (22.8)	1.29 [0.90–1.86]
Preeclampsia or eclampsia			
No	137 (91.3)	793 (92.4)	Ref.
Yes	13 (8.7)	65 (7.6)	1.13 [0.65–1.98]
Maternal complications after delivery*	8 (5.3)	33 (3.8)	1.33 [0.32–5.49]
Length of postpartum hospitalization ^(1)^			
1–3 days	129 (86.0)	773 (90.2)	Ref.
4–6 days	20 (13.3)	66 (7.7)	1.63 [0.96–2.76]
≥ 7 days	1 (0.7)	18 (2.1)	0.37 [0.02–5.43]
Newborn			
Mean (SD) birthweight (g)	3172.72 (556.73)	3109.89 (601.44)	*p* = 0.395; Dif = 62.83 [-120.52–246.19]
Gestational age at birth (weeks)			
< 34	5 (3.3)	35 (4.1)	0.84 [0.07–8.53]
34–36	12 (8.0)	59 (6.9)	1.14 [0.44–2.94]
≥ 37	133 (88.7)	764 (89.0)	Ref.
Adequacy of birthweight to GA ^(2)^			
SGA (birthweight < P10)	18 (12.0)	115 (13.4)	0.91 [0.48–1.70]
AGA (P10 < birthweight < P90)	114 (76.0)	649 (75.7)	Ref.
LGA (birthweight > P90)	18 (12.0)	93 (10.9)	1.09 [0.56–2.10]
Macrosomia (birthweight ≥ 4000g)^(1)^	7 (4.7)	35 (4.1)	1.12 [0.57–2.20
Fetal death	–	3 (0.3)	–
Apgar score– 5^th^ minute < 7^(2)^	3 (2.0)	14 (1.7)	1.16 [0.26–5.20]
Need of intubation^(3)^	2 (1.3)	20 (2.4)	0.60 [0.04–9.14]
NICU admission	29 (19.3)	126 (14.7)	1.32 [0.92–1.89]
NICU indications^†^^(4)^	6 (20.7)	30 (23.8)	1.16 [0.41–3.30]
NICU length of admission^(4)^			
1–3 days	13 (44.8)	51 (40.5)	Ref.
4–6 days	7 (24.1)	16 (12.7)	1.50 [0.29–7.82]
≥ 7 days	9 (31.0)	59 (46.8)	0.65 [0.19–2.19]
Phototherapy for jaundice^(5)^	37 (25.0)	169 (19.9)	1.28 [0.84–1.94]
Major fetal malformation^(6)^	2 (1.3)	13 (1.5)	0.896 [0.31–2.59]
Neonatal Near Miss^‡^^(7)^	8 (5.3)	38 (4.4)	1.18 [0.28–4.89]
Neonatal death	–	7 (0.8)	–
Any adverse neonatal HIP outcome^#^	65 (43.3)	330 (38.5)	1.19 [0.93–1.51]
**Total**	**150**	**858**	

HIP = Hyperglycemia in Pregnancy; GA = Gestational Age; SGA = Small for Gestational Age; AGA = Adequate for Gestational Age; LGA = Large for Gestational Age; NCIU = Neonatal Care Intensive Unit; PPH = Postpartum hemorrhage

*Maternal complications after delivery = Severe sepsis OR Sepsis OR PPH OR Endometritis OR Hysterectomy due to hemorrhage or infection

^†^NCIU indications = respiratory distress or hypoglycemia or asphyxia or congenital abnormality

^‡^Neonatal Near Miss = Apgar 5^th^ min < 7 OR Birthweight < 1750g OR Gestational age < 33 weeks

^#^Any adverse neonatal HIP outcome = Gestational age ≤ 37 weeks OR LGA OR Macrosomia OR Apgar 5^th^ min < 7 OR Need of intubation OR NICU OR Phototherapy for jaundice

^(1)^ Missing = 1

^(2)^ Missing = 43

^(3)^ Missing = 10

^(4)^ Missing = 853 (No NICU admission)

^(5)^ Missing = 11

^(6)^ Missing = 0, after considering 632 missing values as not

^(7)^ Missing = 0, after considering 962 missing values as not

Multivariate analysis showed that a BMI ≥ 26.3Kg/m^2^ [RRadj = 1.87; 1.66–2.10] and a family history of DM [RR_adj_ = 1.71; 1.37–2.15] at study enrollment were independent factors associated with HIP (Table 5).

**Table 5:** Factors independently associated with HIP by multivariate analysis.

Characteristics	RR_adj_ (95%CI)
Body Mass Index at enrollment (overweight or obesity)	**1.87 [1.66–2.10]**
Family history of DM	**1.71 [1.37–2.15]**

- Variables included in the model: outcome is HIP; the predictors are all variables from Tables 2 and 3

## Discussion

In low-risk nulliparous pregnant women included in the Preterm SAMBA Brazilian cohort, the prevalence ofof HIP was14.9% of which 94.7% wasGDM and 5.3% was DIP. A family history of DM, overweight, obesity and previous conditions including polycystic ovarian syndrome (POS), thyroid dysfunctions and hypertensive disorders were identified as factors associated with HIP. However, only a BMI ≥ 26.3Kg/m^2^ and a family history of DM at study enrollment were shown to be independent risk factors for HIP.

The high incidence of GDM in our Brazilian cohort is not surprising. In a recent study conducted in Finland, 16.5% of the nulliparous women evaluated were diagnosed with gestational diabetes [10]. This result is in line with our findings, but lower rates have also been reported. In Ireland and in the United Kingdom the incidence of GDM was 8.9% in nulliparous women at risk and 7.7% in those who are not at risk for the condition. In Australia, only 4.8% of the nulliparous investigated women had GDM [11,12]. The criteria for GDM and HIP, nutritional/diet habits and the characteristics of the population, especially the prevalence of obesity, are the main reasons for the disparities on the prevalence of HIP in the different populations. The incidence of HIP among nulliparous women is of public health concern. It has been estimated that a woman with GDM in her first pregnancy has a 50% risk of GDM recurrence in her second pregnancy [19]. Since Brazil is one of the eight countries responsible for 55% of deliveries and 55% of diabetes cases worldwide [1], having a GDM rate of 14.9% in nulliparous women makes matters even worse. Thereby, there is an urgent need for the early prediction, diagnosis and treatment of HIP.

Maternal age, ethnicity, pregestational BMI, family history of DM, previous GDM and macrosomia, multiparity and hypertension are well-established clinical risk factors for GDM [7–9,20]. However, the prevalence of overweight and obesity epidemic in women of childbearing age contributes to a higher risk of GDM [20,21–24]. Only few studies have actually reported risk factors for GDM in nulliparous women [10–12,25]. In Brazilian nulliparous women, these risk factors have still not been published. Therefore, our study may help to address this deficit.

Risk-based screening is controversial. While some authors consider this type of screening inadequate and inconsistent, others support that offering an OGTT to women aged ≥ 25 years old and/or with a BMI ≥ 26.3kg/m^2^ is as effective as more complex risk prediction models [9,26,27–29]. In our nulliparous Brazilian cohort, overweight or obesity and a family history of DM were independent risk factors for HIP.

In our cohort study, a family history of DM occurred in 31.3% of HIP and in 17.7% of the control group. These rates thus differed between pregnant women with and without GDM, and were lower than previously published–rates of about 40 to 50% in the GDM group and 35 to 40% in the control group [12,29]. This finding may contribute to the discrepancy between our results and other reports in the literature.

In Brazilian nulliparous women, a BMI ≥ 26.3Kg/m^2^ at 19 to 21 weeks of gestation should be highlighted. In our study, the risk of developing HIP increased almost twofold in overweight or obese women. Irrespective of parity, a systematic review showed that the risk for GDM rises progressively according to BMI category [30]. Several studies have previously shown an association between the degree of maternal adiposity and hyperglycemia, while others have identified that maternal age is the modulating factor [17,20–23,30–32]. In nulliparous women, some recent studies also demonstrated the same association [10–12,24,25]. Therefore, the current literature supports our assertion that overweight or obesity before 21 weeks of gestation is an independent risk factor for the development of HIP (GDM or DIP) in nulliparous Brazilian women.

In general, maternal glucose control began with medical nutrition therapy (MNT), physical activity, and weight control. Insulin treatment was initiated whenever glycemic goals (FPG < 95 mg/dL and a 2h postprandial glucose test < 120 mg/dL) were not met through MNT and regular exercise [5,33]. Although evidence supports the efficacy and short-term safety of oral anti-diabetic agents, these drugs do cross the placenta and data on long-term effects are lacking [34]. The Brazilian drug safety regulatory agency (ANVISA) has not yet released the oral anti-diabetic agents for use in pregnancy. Insulin would be the pharmacological option for maternal glucose control in Brazil.

In our study, about 40% (58/150) of the HIP cases did not receive treatment. Of those treated, a lower proportion of women (21.7%) received diet and exercise, and pharmacological therapy. Both insulin and metformin were the predominant drugs indicated (78.3%). Regardless of this unsatisfactory scenario, perinatal results were statistically similar in HIP and control groups. Nevertheless, these results were unexpected. The limited number of cases in the HIP group, and diagnostic criteria used, in association with the high prevalence of overweight and obese women in the control group may explain this issue.

According to the literature, all these possibilities could mask the expected differences in perinatal outcomes. In IADPSG protocol studies, the small sample size was used to explain the increasing incidence of GDM (10 to 25%) and limited effect on perinatal results [35–38]. Overweight or obesity alone has been associated with the risk of developing GDM and Metabolic Syndrome (MetS). HOMA-IR levels increased, producing a pronounced effect on excessive fetal growth, irrespective of maternal glucose status [22,39,40]. Thus, bias could occur in perinatal outcomes in non-diabetic pregnancies.

### Strengths and limitations

In our study we evaluated healthy nulliparous Brazilian women in a prospective multicenter cohort from five public maternity hospitals, corresponding to a multi-regional and mixed population in an upper-middle income country. In addition, our study highlighted some problems in the quality of diabetes care in pregnancy. Our results may contribute to the identification of risk factor for HIP in nulliparous women which were not done previously worldwide, especially in Brazil where pertinent data are not available.

Our study has some limitations. Pregestational weight records are lacking, the sample size was not specifically calculated for HIP outcomes and glucose control was not standardized in collaborating centers. Nevertheless, our study reflected local protocols and the reality of obstetric referral centers.

To the best of our knowledge, this was a pioneer study in Brazil. Other studies with a larger sample size may confer increased statistical power to the results and identify new risk factors for hyperglycemia in healthy nulliparous pregnant women.

## Conclusions

There was a high incidence of HIP (14.9%) in a nulliparous Brazilian cohort, with 94.7% of the cases due to GDM. A family history of DM, overweight or obesity and some previous clinical conditions were associated with HIP. However, only a BMI ≥ 26.3Kg/m^2^ at study enrollment and a family history of DM were shown to be independent risk factors for HIP. While there is no incontrovertible evidence to support universal screening, a family history of DM and a BMI ≥ 26.3Kg/m^2^ (until 19–21 weeks of gestation) may be used as selective markers for Brazilian nulliparous women. This strategy will potentially ameliorate the diagnostic performance of HIP in low-resource settings where universal screening is not easily available. Taking into account the scarcity of results in nulliparous women, our findings may contribute to determine the optimal diagnostic approach to HIP in Brazil and in other countries with similar socioeconomic characteristics.

## Supporting information

S1 Data (XLSX)
